# Current Status and Family Factors Influencing Caries in the Deciduous Teeth of Children 3–6 Years of Age in Families Residing in Rural Heishanzui Township

**DOI:** 10.3290/j.ohpd.b5245819

**Published:** 2024-04-23

**Authors:** Liming Zhang, Yaxuan Liu, Ruiming Chu, Yan Zhao, Bing Liu, Chunguo Fan, Peng Song

**Affiliations:** a Dentist, Department of the Second Hospital of Shijiazhuang, Shijiazhuang City, China. Study design, collected and analysed the data, wrote the manuscript.; b Dentist, Department of the Second Hospital of Shijiazhuang, Shijiazhuang City, China. Performed the interviews, collected the data, wrote the manuscript.; c Public Health Official, Department of Manchu Autonomous County of Fengning Heishanzui Town Central Hospitals, Chengde, Hebei, China. Study design, study administration, data collection.; d PhD Student, Hebei Medical University, Hebei, China. Data collection and analysis, critically edited the manuscript.; e Public Health Official, Department of School and Hospital of Stomatology, Hebei Medical University, Hebei, China. Assisted in the study design and data collection.; f Dentist, Department of School and Hospital of Stomatology, Hebei Medical University, Hebei, China. Performed the interviews, analysed the data, wrote the manuscript.; g Dentist, Department of Oral Pathology, School and Hospital of Stomatology, Hebei Medical University, The Oral Disease Clinical Medical Research Center of Hebei, Hebei, China. Study design, statistical analysis, wrote the manuscript.

**Keywords:** caries, children, deciduous dentition, oral health behaviours, rural areas

## Abstract

**Purpose:**

To determine the caries status in children’s deciduous teeth and examine the influence of family oral health behaviours on the caries status.

**Materials and Methods:**

This cross-sectional study included 329 children aged 3–6 years in rural Heishanzui Township, Hebei Province, China, and used a completely random sampling method. These children underwent physical and oral health examinations. The questionnaires were given to the parents and caregivers of the examined children.

**Results:**

The prevalence of caries in the deciduous dentition among children aged 3–6 years was 80.55%, with a dmft index of 4.93. Children in the caries group ate sweets, chocolates, and carbonated drinks more frequently than did children in the caries-free group (p < 0.05). Children in the caries-free group brushed their teeth more frequently, with parents helping their children brush, more often than did those in the caries-affected group (p < 0.05). The level of parental education and annual household income also had statistically significant effects on the prevalence of caries in the two groups (p < 0.05). Logistic regression analysis revealed that the frequency of eating sweets was a risk factor for caries in deciduous teeth (odds ratio = 2.20; p < 0.05).

**Conclusion:**

The prevalence of caries in deciduous teeth among children aged 3–6 years in rural Heishanzui Township was high. Compared to children in the caries-affected group, the families and children in the caries-free group had better oral hygiene behaviours. Moreover, the frequency of eating sweets was shown to be a risk factor for caries in deciduous teeth in children aged 3–6 years.

Caries is one of the most common oral diseases in children. According to a meta-analysis, the global prevalence of dental caries among children from 1995–2019 was 46.2% (n = 80,450) for deciduous teeth and 53.8% (n = 1,454,871) for permanent teeth.^4^

Severe carious lesions have a major impact on the normal development of permanent teeth in preschool children by causing masticatory function disorders.^5^ Indeed, the severity of caries may lead to pedatrophy (severe malnutrition) and delayed growth.^5^ Dietary factors are crucial in the pathogenesis of caries. It has been shown that sweet foods considerably increase the extent of caries and that refined, sticky, sugary foods have a high cariogenic potential.^6,15^ Therefore, the relationship between oral health behaviours and caries in children is a current research focus.^9,15^ Caries prevalence has been reported to be lower among children with the following: infrequent consumption of sweets, parents who have a higher level of education, and maternal oral health education.^9,14^ Higher parental oral health literacy promotes oral health in children.^18^ Several studies have shown that parental employment status, age, and sex are also strongly correlated with dental caries in children.^1^ According to a Brazilian study, poor children have a higher rate of caries,^10^ which may be related to family factors and parental eating habits.

According to a recent study, dental health services for preschool children in Northwest China were limited in the last decade.^3^ The utilisation rates of dental healthcare services were 20.8% and 20.0% in 2005 and 2015, respectively, and were even lower in rural areas, where caregivers in rural families have only superficial knowledge of the oral health status of their children.^3^ According to the 2019 Fourth Oral Health Epidemiology Survey,^7^ the prevalence rate of caries in deciduous teeth among 5-year-old children was 70.9%, which has increased compared with that 10 years earlier. The prevalence rate was greater in rural than in urban areas.^7^ Heishanzui (Fengning Manchu Autonomous County, Chengde city, Hebei Province, China) is a rural mountainous town in northwestern China that is predominantly inhabited by people of Manchu ethnicity; only a small part of the population belong to other ethnicities. The diet and cultural practices of the Manchus in Heishanzui are ethnic. Reports pertaining to caries, oral health status, and associated factors in children who reside in Heishanzui are incomplete.

The present study was conducted with the implementation of the Spring Rain Project Help Scheme in rural Hebei Province to investigate and analyse the prevalence of caries, family oral health behaviours, and factors influencing these parameters in children aged 3–6 years in Heishanzui Township.

## Materials and Methods

### General Information

A total of 329 3- to 6-year-old children from rural families residing in Heishanzui Township were investigated using a complete random sampling method based on the Fourth National Oral Health Epidemiological Survey Programme^7^ following the principles of economy and efficiency. The children underwent a general physical examination with an oral health examination by a stomatologist, and oral health behaviour questionnaires were completed by the parents/caregivers of the children with instruction by a questionnaire surveyor.

This study was approved by the Medical Ethics Committee of the Hospital of Stomatology, Hebei Medical University (No. 2021-067). The parents/caregivers of the included children signed written informed consent forms before the examinations and the survey.

### Survey Methodology

The caries test criteria listed in the Basic Methods of Oral Health Survey and the Fourth National Oral Health Epidemiological Survey published by the WHO^7^ were used to determine the caries status of children aged 3–6 years.

The questionnaire was administered face-to-face on site by a questionnaire surveyor, one of three medical staff members affiliated with a local health-service accredited organisation. The questionnaire surveyor instructed the father, mother, or caregivers of the child to complete the paper-based questionnaire, which was collected on site. A total of 329 questionnaires were distributed, and 329 valid questionnaires were returned (response rate = 100%). The general physical and oral health examinations were conducted by stomatologists. Before the survey, the stomatologists and questionnaire surveyors underwent uniform training. Standard consistency tests were performed before and during the survey, and the kappa values of intra-rater reliability at the two testing times was > 0.8. The examination sites were located at village clinics in Heishanzui Township, Hebei Province, and in the Department of Manchu Autonomous County of Fengning Heishanzui Town Central Hospital. The examination conditions at the survey sites were consistent, with uniformly configured dental mouth mirrors, probes, and community periodontal index (CPI) probes. A technical steering group consisting of experts from departments at the Stomatology Hospital of Hebei Medical University who had extensive clinical experience in endodontic and restorative dentistry, prophylaxis, and oral surgery was responsible for survey quality control. In addition, the technical steering group guided and trained the medical staff of the local Heishanzui Township Central Health Center and the local village stomatologists in implementing the survey.

### Statistical Methods

SPSS 23.0 software was used to analyse the caries status of deciduous dentitions. A *χ*^2^ test was applied to test the differences in caries prevalence, the mean number of carious lesions, the decayed, missing and filled teeth (dmft) index for each item, the general situation of the families, and the differences in oral health behaviours in the children. The dependent and independent variables were defined, and the trend *χ*^2^ test was used to analyse the caries risk factors. The factors influencing the dependent variable were preliminarily screened, and multifactorial analysis was subsequently performed to establish a logistic regression model to analyse the relationship between caries and related factors. The odds ratio (OR) and 95% confidence interval (CI) were calculated.

## Results

### Caries Status of Deciduous Teeth

In the present study, 265 of the 329 children aged 3 to 6 years in Heishanzui Township were examined for caries in deciduous teeth. Caries prevalence in deciduous teeth was 80.55% and the dmft index was 4.93. The prevalence of caries among children in different age groups varied slightly. Caries prevalence and the dmft index differed slightly with increasing age, and the difference in caries prevalence between the age groups was not significant (*χ*^2^ = 5.181, p > 0.05; Table 1). One hundred fifty-two boys were examined. Of the boys, 126 had caries, with a caries prevalence of 82.89%. Of the 177 girls examined, 139 had caries, with a prevalence of 78.53%. The difference between the caries prevalence in boys vs girls was not statistically significant (p > 0.05; Table 1). Children from Fengning Manchu Autonomous County (Chengde city) accounted for the majority of the population; there were also children from other ethnic minority groups. Ethnicity was analysed as an item. The prevalence of caries among children of different ethnicities was as follows: Manchu children (223 of 267) had caries with a prevalence of 83.52%; Han children (26 of 37) had caries with a prevalence of 70.27%; and Mongolian children (13 of 21) had caries with a prevalence of 61.90%. Three of 4 children in the other ethnic groups had caries (75.0%). The difference in caries prevalence among the ethnic groups was not statistically significant (p > 0.05; Table 1).

**Table 1:** Prevalence of caries in deciduous teeth among rural children aged 3–6 years (%)

Variable	Sex		Ethnicity				Age (years)			
	Male	Female	Manchu	Han	Mongolian	Other	3	4	5	6
N	152	177	267	37	21	4	142	86	79	22
Caries	126	139	223	26	13	3	118	62	67	18
Caries free	28	38	44	11	8	1	24	24	12	4
Caries prevalence (%)	82.89	78.53	83.52	70.27	61.90	75.00	83.10	72.09	84.81	81.82
X^2^	0.474 p = 0.491		7.787 p = 0.051				5.181 p = 0.159			
p										
dmft (M)							4.86	4.97	5.12	4.78

### Analysis of Oral Health Behaviours among Rural Families with Children

#### Dietary behaviours

Children with caries statistically significantly more frequently consumed desserts, sweets, chocolates, carbonated beverages, fruit juices, and sweetened dairy products than did children without caries (*χ*^2^ test, p < 0.05). There was no statistically significant difference in eating sweets at bedtime between children with and without caries (p > 0.05; Table 2).

**Table 2:** Relationship between eating behaviours and caries in children aged 3–6 years (%)

Variable	Desserts (bread, cakes) (times/week)			Candy/chocolate (times/day)			Carbonated drinks (times/day)			Juice/flavoured dairy products* (times/day)			Consumption of sweets before bedtime	
	≥7	1–6	<1	≥7	1–6	<1	≥7	1–6	<1	≥7	1–6	<1	Yes	No
N	126	160	43	87	198	44	64	232	33	142	166	21	132	197
Caries	101	143	21	84	176	5	58	192	15	122	134	9	128	137
Caries free	25	17	22	3	22	39	6	40	18	20	32	12	4	60
Caries prevalence (%)	80.16	89.38	48.84	96.55	88.89	11.36	90.63	82.76	45.45	85.92	80.72	42.86	96.97	69.54
X^2^		35.565			157.423			30.809			17.334		1.471	
p		0.01			0.01			0.01			0.01		0.225	

*Flavoured dairy products include flavoured milk drinks and sweetened flavoured yogurt.

#### Brushing behaviours

Children without caries brushed their teeth more times per day than did children with caries. Moreover, the parents of children without caries helped their children brush their teeth more frequently (p < 0.05). The age at which children with or without caries began brushing their teeth had no effect on the development of caries (p > 0.05; Table 3).

**Table 3:** Relationship between toothbrushing behaviours and caries in rural children aged 3–6 years (%)

Brushing behaviours	Age at start of brushing (years)			Number of times per day			Parental help with brushing		
	≤3	4–5	Occasionally/never	≥2	1	<1	Daily	Weekly	Occasionally/never
N	102	226	1	88	226	15	39	79	211
Caries	78	186	1	59	191	15	8	65	192
Caries free	24	40	0	29	35	0	31	14	19
Caries prevalence (%)	76.47	82.30	100	67.05	84.51	100	20.51	82.28	91.00
X^2^		1.917			17.814			104.559	
p		p = 0.413			p < 0.01			p < 0.01	

#### Comparison of family income

The parents of children with and without caries were compared with respect to education level and annual family income. The differences were statistically significant (p < 0.05; Table 4), where higher education and income were associated with lower caries prevalence.

**Table 4:** Relationship between parental education/ annual household income and caries in rural children aged 3–6 years (%)

Variable	Head of the household			Annual household income (RMB, yuan)		
	Junior high school and below	High school or polytechnic school	University and above	20,000 and above	20,000–50,000	50,000 and above
N	201	109	19	186	134	9
Caries	188	74	3	172	91	2
Caries free	13	35	16	14	43	7
Caries prevalence (%)	93.53	67.89	15.79	92.47	67.91	22.22
X^2^		74.462			47.150	
p		p < 0.01			p < 0.01	

#### Logistic regression analysis of factors associated with caries in deciduous teeth

A type of generalised linear regression analysis (logistic regression analysis) was performed with caries prevalence as the dependent variable and five influencing factors (frequency of eating sweets, number of times teeth were brushed per day, whether parents helped children brush their teeth, level of parental education, and annual family income) as the independent variables. The results are shown in Table 5; the frequency of eating sweets had a statistically significant effect on caries prevalence (OR = 2.20; p < 0.05).

**Table 5:** Logistic regression analysis of factors associated with caries in rural children aged 3–6 years

Selected variable	Regression coefficient B	Standard Error SE	Wald X^2^	p	OR and 95% confidence interval
Frequency of eating sweets	2.132	0.236	10.236	0.000	2.20 (2.18–2.38)
Number of times per day teeth are brushed	-1.414	0.137	8.343	0.000	0.28 (0.24–0.56)
Parents help children brush their teeth	-1.028	0.426	6..568	0.012	0.29 (0.27–0.66)
Parental education level	-0.348	0.755	7.456	0.023	0.31 (0.35–0.72)
Annual family income	-1.563	0.645	10.132	0.000	0.34 (0.29–0.63)

## Discussion

The results of this investigation showed that caries prevalence in deciduous teeth among children aged 3–6 years in Heishanzui Township was 80.55% and the dmft index was 4.93. These values are higher than those for caries prevalence and dmft index in Guizhou^8^ and Chengdu city, Sichuan Province,^19^ Chongqing,^17^ and Xi’an.^16^ We also found a correlation between caries prevalence and geographic and ethnic differences. However, caries prevalence in various ethnic groups did not differ statistically significantly (p > 0.05). Nevertheless, the prevalence of caries among Manchu children and children of other ethnic minorities was very high, and many of the affected teeth were not treated in a timely manner. After quantitative grading of factors related to caries in rural children in Heishanzui Township and performing a *χ*^2^ test for trend, it was shown that the frequency of eating sweets, number of times children brushed their teeth per day, whether parents helped their children brush their teeth, parental education level, and annual family income were related to caries prevalence in deciduous teeth.

The oral cavity is an ecosystem of different structures, tissues, and complex microbial communities. A high frequency of eating sweets can perpetuate an acidic state in the oral cavity with a low plaque pH, which can demineralise the enamel, leading to dental caries.^12^ Parents who are not vigilant or lack adequate awareness of oral-health care may be an important factor with respect to the increased consumption of sweets by children. An increased consumption frequency of desserts (breads and cakes), sweets, chocolates, carbonated beverages, fruit juices, and flavoured dairy products (flavoured milk drinks and sweetened flavoured yogurt) contributed to higher caries prevalence. The present survey showed that the frequency of eating sweets, such as candies, chocolates, and carbonated drinks, was high among all children residing in Heishanzui Township. The intake of sugar-containing foods by rural children should be limited as much as possible in the future.

Family oral health behaviours play an important role in caries prevention.^9,14,18^ The present investigation revealed that an increase in the frequency of daily toothbrushing and the presence of parental guidance and supervision of toothbrushing significantly reduced the caries prevalence, and these differences are consistent with those in previous reports.^9,14,18^

Childhood caries is strongly associated with socioeconomic and family factors.^1,10,11^ As shown in Tables 4 and 5, parental education and annual family income were related to the prevalence of childhood caries. According to national and international studies,^1,10,11,20^ adequate family economic status and a higher level of parental education lead to greater attention given to children’s oral health, so that the proportion of children with caries is correspondingly lower. Saethre-Sundli et al^13^ also showed that socioeconomic status is negatively correlated with the dmft index in children.

This study showed that awareness of oral-health care was low among parents, indicating that oral health promotion and education are inadequate. Therefore, oral hygiene knowledge and education should be further strengthened to improve parents’ own oral hygiene behaviour, so that they can monitor and improve their children’s oral hygiene. Public-health nurses/dentists can be very effective at improving children’s oral health. Indeed, barriers to the promotion of oral health encountered by public-health nurses/dentists should be taken into account. In addition, public-health nurses/dentists and teams can improve children’s oral health by enhancing preventative oral health care, training oral health facilitators, and addressing associated barriers.^2^

This study was limited by its small sample size from only one regional rural area. Additional samples from similar areas need to be investigated to determine the prevalence and factors influencing childhood caries in rural areas. Moreover, the juice and flavoured dairy products were put in the same category in the questionnaire, which may have caused response bias.

## Conclusion

The rural area of Heishanzui Township, China, had a high prevalence of caries in deciduous teeth in children aged 3–6 years. Compared to children with caries, the families and children in the caries-free group had better oral hygiene behaviours. The frequent consumption of sweets was shown to be a risk factor for caries in deciduous teeth in children aged 3–6 years in Heishanzui Township.
